# Eggs in the Freezer: Energetic Consequences of Nest Site and Nest Design in Arctic Breeding Shorebirds

**DOI:** 10.1371/journal.pone.0038041

**Published:** 2012-06-12

**Authors:** Ingrid Tulp, Hans Schekkerman, Joep de Leeuw

**Affiliations:** 1 Institute for Marine Resources and Ecosystem Studies, IJmuiden, The Netherlands; 2 Sovon Dutch Centre for Field Ornithology, Nijmegen, The Netherlands; 3 Institute of Freshwater Research, Swedish University of Agricultural Sciences, Drottningholm, Sweden; Arizona State University, United States of America

## Abstract

Birds construct nests for several reasons. For species that breed in the Arctic, the insulative properties of nests are very important. Incubation is costly there and due to an increasing surface to volume ratio, more so in smaller species. Small species are therefore more likely to place their nests in thermally favourable microhabitats and/or to invest more in nest insulation than large species. To test this hypothesis, we examined characteristics of nests of six Arctic breeding shorebird species. All species chose thermally favourable nesting sites in a higher proportion than expected on the basis of habitat availability. Site choice did not differ between species. Depth to frozen ground, measured near the nests, decreased in the course of the season at similar non-species-specific speeds, but this depth increased with species size. Nest cup depth and nest scrape depth (nest cup without the lining) were unrelated to body mass (we applied an exponent of 0.73, to account for metabolic activity of the differently sized species). Cup depth divided by diameter^2^ was used as a measure of nest cup shape. Small species had narrow and deep nests, while large species had wide shallow nests. The thickness of nest lining varied between 0.1 cm and 7.6 cm, and decreased significantly with body mass. We reconstruct the combined effect of different nest properties on the egg cooling coefficient using previously published quantitative relationships. The predicted effect of nest cup depth and lining depth on heat loss to the frozen ground did not correlate with body mass, but the sheltering effect of nest cup diameter against wind and the effects of lining material on the cooling coefficient increased with body mass. Our results suggest that small arctic shorebirds invest more in the insulation of their nests than large species.

## Introduction

Most birds build a nest to lay and incubate their eggs in. The possible functions of building a nest can be various [1]. It might simply serve to keep the eggs together and keep individual eggs from rolling away [2], thus reducing the risk that one or more eggs are not incubated properly. A nest can also provide protection against predation [3], [4]. A well hidden nest in a deep scrape, perhaps even concealed partly by vegetation, is likely to reduce predation risk, not only if the bird sits on the nest, but also in its absence. A lined nest scrape can also substantially reduce the rate at which the eggs lose heat and enable the parents to control humidity inside the nest [5], [6]. Heat conservation is particularly important in cold environments [7]. Additionally the energy expenditure of the incubating adult bird can be reduced because of the insulative properties of the nests [8].

The regulation of egg temperatures can be energetically demanding for parent birds [9]. Energy is required to maintain the temperature of the eggs at an appropriate level to ensure embryo development and to rewarm clutches that cooled down during the parents’ absence [9]. In the Arctic, where daily energy expenditure is elevated because of the cold environment, incubation is costly, particularly for small shorebirds (Charadrii) [10], [11], [12]. Selection should therefore favour nest designs that reduce the rate of heat loss as much as possible in the light of other factors such as nest predation risks [13], [14]. The majority of shorebirds breed on the ground. They lay their eggs in nest cups varying from none at all (e.g. coursers, Glareolidae), a shallow hole without any nest lining (e.g. Kentish plover *Charadrius alexandrinus*), to rather deep and thickly lined scrapes (e.g. redshank *Tringa tetanus* [15]), sometimes hidden in thick vegetation but more often in more open sites such as grasslands and sparsely vegetated open ground [16], [17]. Shorebirds generally lay pointed eggs. The position of the eggs oriented with their pointed ends towards the centre and downwards minimizes the amount of space needed to form the nest and increases the efficiency of the heat transfer from parent to egg. Most shorebird nests consist of scrapes that are made by one of the mates by pushing their breast towards the ground and scraping bottom surface material with their feet, using their breast to round the nest edges. The scrape is lined with a variety of materials including grass, moss, lichens or grit, forming a simple structure with a limited amount of lining material compared to nests of many other birds.

Many shorebird species breed in arctic regions, often nesting on open tundra just a few decimetres above the permafrost. [18] experimentally showed that in eggs of pectoral sandpipers *Calidris melanotus* placed in an excavated scrape and in a scrape with nest lining added, heat loss rates were reduced by 9% and 25%, respectively, in comparison with eggs placed on the tundra surface. This suggests that lined scrapes improve the insulation of clutches. They also showed that the insulative properties of a nest are determined by nest cup depth and shape, the thickness of the lining, and the type of lining material [18]. Furthermore, ground temperature has been shown to have an important effect on heat loss to the ground [19]. In nests of pectoral sandpiper that were experimentally heated, nest attendance increased, the effect being stronger when ground temperature was lower.

Piersma et al. (2003) showed that shorebirds incubating clutches in high arctic tundra have a Daily Energy Expenditure (DEE) that is about 50% higher than that of similarly sized birds breeding in temperate areas. The allometric scaling exponent for DEE was 0.55, which is smaller than the scaling exponents for Basal Metabolism (0.73–0.71, Lasiewski and Dawson 1967; Lindström and Klaassen 2003), and for maximum sustained levels of energy turnover in birds (0.72, Kirkwood 1983; Kvist and Lindström 2000). Consequently, DEE during incubation is likely to represent a larger challenge to the energy-processing capacity of small than larger species, and small species will have most to gain by reducing heat loss from nests. We therefore hypothesise that within the same environment, small shorebirds should place their nests either in more thermally favourable microhabitats, or invest more in nest insulation than larger species. In addition to this body size effect, parental care system may play a role because species with uniparental incubation have less time available for foraging than species which share incubation duties roughly equally between the sexes, even while their nests are unattended during a greater proportion of time [12], [20], [21]. A well-insulated nest may be important in these species to reduce egg cooling rates and increase the potential length of feeding absences.

We tested the hypothesis that small species place their nests in more thermally favourable microhabitats and/or invest more in nest insulation than large species, by collecting data on nest location, nest cup size and shape, and thickness and composition of lining material in six shorebird species breeding sympatrically in the arctic tundra of western Taimyr, Siberia, Russia. We applied the quantitative relationships between nest properties and egg cooling coefficient derived for pectoral sandpiper nests by [18] to estimate their relative effect in these six species, singly and in combination.

## Methods

### Study Area and Species

Permission to work in the Great Arctic reserve was given by its director prior to the fieldwork. Data were collected during June-early August 2002 at Medusa Bay, in the west of the Taimyr Peninsula, Siberia, Russia (73°20′N, 80°30′E). The habitat consists of arctic tundra [22], characterised by rolling hills up to 50 m above sea level, and scattered stony ridges. Vegetation consisted of moss, lichen, grasses and polar willows *Salix polaris* generally not higher than 10 cm, with a significant proportion of the soil surface bare. Sedge meadows with low *Salix reptans* shrubs occur in wet valleys and in flat places on the watersheds. Average summer temperature (2000–2002) and wind speed in the incubation period (ca 15 June–15 July) is 4.3°C and 7.1 ms^−1^. A more detailed description is provided elsewhere [23], [24].

We collected data on nests of six shorebird species (ordered by increasing average mass during incubation as measured in the study area [25]: little stint *Calidris minuta* (30 g, N = 61 nests), red phalarope *Phalaropus fulicarius* (51 g, N = 6), dunlin *Calidris alpina* (54 g; N = 22), curlew sandpiper *Calidris ferruginea* (65 g; N = 12), ruddy turnstone *Arenaria interpres* (101 g; N = 9), and Pacific golden plover *Pluvialis fulva* (133 g, N = 18). These species were the most common breeding species in the year of study. Common ringed plover *Charadrius hiaticula* is also a common breeding bird in the area but was excluded from this study because it nests in a very different habitat (gravel plains and shingle banks along rivers). Although the six species did show differences in their preferred nesting habitat within the vegetated tundra (with red phalarope, little stint and dunlin preferring the wetter areas and curlew sandpiper, turnstone and Pacific golden plover the dryer parts), there was extensive overlap between them and nests of different species were often found in close proximity. Incubation is uniparental in little stint, red phalarope and curlew sandpiper, and is shared between the sexes in the three other species [15], [26], [27], [28].

### Nest Measurements

Shorebirds started laying eggs shortly after snow melt in mid June. Nests were located by intensive searching during and after the laying period. When a nest was found we categorised its general position: on horizontal ground either in lowlands or on ridge tops, or on slopes facing roughly north, south, east or west. These positions were given a rank score with respect to thermal favourability on the basis of their exposure to sun (favourable) and wind (unfavourable). In northern Taimyr in summer, northern winds are generally cold since they arrive over the sea-ice of the Arctic Ocean; southern winds bring warmer air from the continent. Nest positions were ranked in decreasing order of favourability as: 1 south slopes, 2 west and east slopes, 3 flat lowlands, 4 flat ridge tops, and 5 north slopes. There was no digitized map with a sufficiently small scale available for this remote area. The distribution of each of these habitats were drawn in by hand on hard copy maps of the study area. The proportional availability of tundra in each of these categories was calculated from these maps using a overlaid grid.

**Figure 1:**
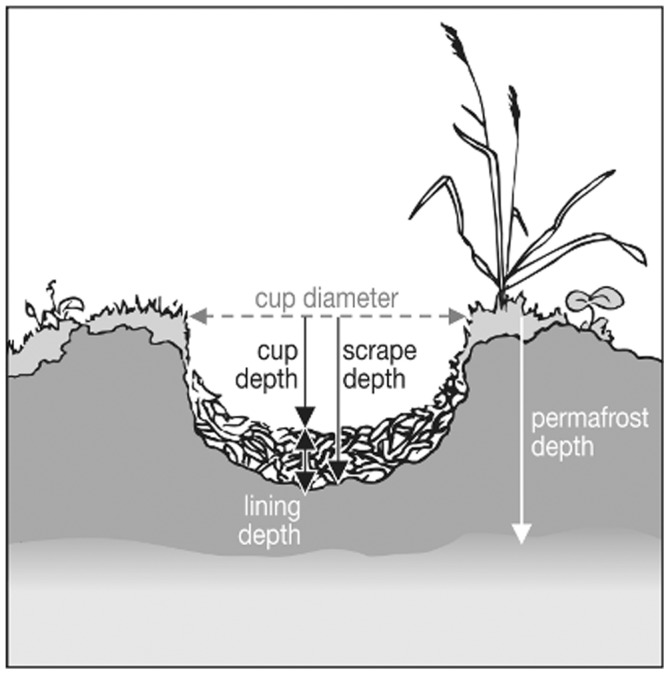
Illustration of nest cup measurements.

Upon finding a nest we floated two eggs in water to estimate the time they had been incubated [25], [29] and back-calculated the laying date (of the last egg). We measured the depth to the frozen ground next to the nest by pushing a metal pin into the substrate until it hit the ice (Fig. 1). Nests were marked using GPS and checked regularly. On at least one of these repeated visits the depth to the frozen ground was measured again. The change in this depth was described by linear regression on all measurements taking into account possible differences between species, and the results were used to estimate the depth to frozen ground at laying for each nest.

**Figure 2:**
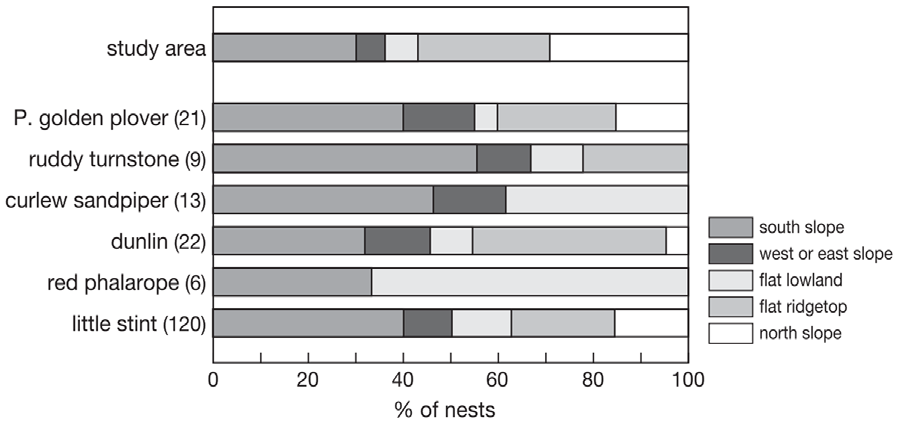
Frequency distribution of breeding sites for six shorebird species, with number of nests in brackets. The upper bar illustrates the relative occurrence of the different categories in the study area.

The depth of the nest cup (cm) was measured by lowering a ruler vertically to the lowest part of the nest cup, placing a second ruler horizontally bridging the opposite edges of the scrape, and reading the depth at their intersection (Fig. 1). Nest cup diameter (cm) was measured with the horizontal ruler in two directions perpendicular to each other (as most cups were slightly oval). The shape of the nest cup (shallow/deep and wide/narrow) was expressed as the depth of the nest cup divided by the surface area ( =  cup depth/diameter 1×diameter 2). The nests were revisited after they were vacated by the birds (clutches hatched or predated). Nest cup depth was measured again and the nest lining was collected into a small plastic bag. The depth of the empty scrape (cm) was measured after removal of the nest lining. The thickness of nest lining (cm) was calculated by subtracting nest cup depth from scrape depth (Fig. 2).

The collected lining material was dried in open plastic beakers near the central heating system in the field station and weighed every two days using a mass balance to the nearest gram, until mass did not decrease between consecutive weighings. Per nest we measured total (dry) mass (g) of the nest lining, its total volume (cm^3^, based on height in the beaker after drying and gentle shaking), and estimated visually (in c. 10% classes) the relative contribution to the total volume of different types of lining material: willow leaves (*Salix polaris* or *S. reptans*), *Thamnolia vermicularis* (a lichen forming loose white filamentous thalli), other lichens, sedge/grass leaves and stems, moss, and other materials.

### Approximating Insulative Properties of Nests

Newton’s law of cooling states that a heated object (in this case an egg) cools down to ambient temperature according to T_egg_  =  T_a_+(T_i_-T_a_)exp(-C x time) with T_i_ and T_a_ the initial and final temperatures of the egg respectively (°C) and the exponential cooling coefficient C (s^−1^) depending on the thermal properties of the object and its environment. Based on this principle, [18] measured the insulative properties of pectoral sandpiper nests by determining C from the cooling curve of pre-warmed clay eggs placed in them, and quantified the relative contribution of several nest features. They found that in deeper nests eggs lose more heat to the surrounding soil, but at the same time they are more sheltered from the cooling effect of wind. A thicker lining reduces heat loss, while the insulative performance varies between types of lining material and decreases when the material is wet.

We used the quantitative relationships derived empirically by [18] to reconstruct the effect of these factors on the egg cooling coefficient for every nest of the six species in our study based on their dimensions and lining composition. We did this by estimating the proportional difference in C between a nest with the measured properties and a nest with average properties of pectoral sandpiper (nest cup depth 3.1 cm, diameter 9.1 cm, lining depth 2.1 cm, lining material 50% grass, 30% leaves, and 20% lichens). Our aim was not to derive a precise prediction of the cooling rate of eggs in our nests, but to be able to compare and combine the effects of different nest features in a way that is consistent with heat loss theory.

**Figure 3:**
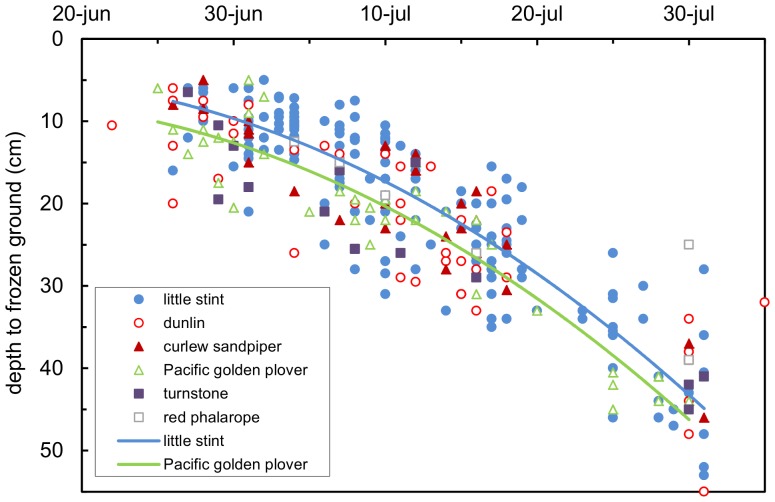
Depth of the frozen ground in relation to depth in six shorebird species. The regression lines for the smallest and largest species are given.

Eggs in deeper nest cups are closer to the permafrost and therefore surrounded by colder soil, which increases heat loss to the ground. To estimate this effect of nest cup depth we used figure 2 of [18]. For nest cup depth ≤3.1 cm the egg cooling coefficient did not depend on cup depth; in the range 3.15 to 7 cm, C increased by 0.64×10^3^ s^−1^ per cm depth. On the other hand, deeper nest cups are better protected from wind as illustrated by the fact that the gradient of the wind speed vs. cooling coefficient relationship declined significantly with increasing scrape depth. [18] worked with nests of a single species and used cup depth as the predictive variable, but we compare nests of different species varying not only in depth but also in diameter. We assumed that the cooling effect of wind is proportional to the ratio of the surface of the nest cup-air interface and nest cup depth. Therefore, we rescaled [18]’s figure 3 predicting the gradient between surface wind speed and egg cooling coefficient using (cup depth/diameter^2^) as the predictor variable instead of cup depth. This yields the equation: gradient  =  (0.29−0.29×(cup depth/diameter^2^))×10^3^.

**Figure 4:**
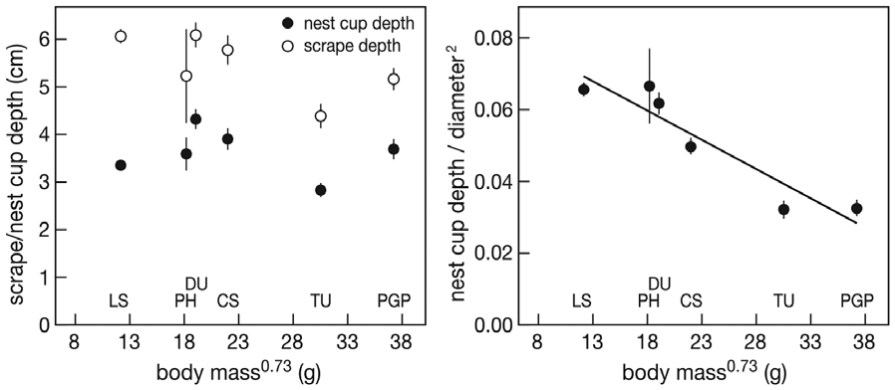
Nest cup and scrape depth (left) and nest cup depth/diameter^2^ (right) in relation to body mass^0.73^. LS  =  little stint, PH  =  red phalarope, DU  =  dunlin, CS  =  curlew sandpiper, TU  =  turnstone, PGP  =  Pacific golden plover. Average and SE values are represented for each species. The line represents the regression line.

Egg cooling coefficient (due to heat loss to the ground) decreases nonlinearly with lining depth, with the strongest reduction when lining depth increases from 0–2 cm but little extra effect of a thicker layer ([18], Fig. 4). The relationship between lining depth and egg cooling coefficient was described by: C = 3.1+7×exp(−1.3×lining depth) (we refitted the relationship in Fig. 3 in [18], as the equation provided in the figure caption contained an error). Cooling coefficients also varied significantly between eggs surrounded by different dry materials and increased in the order: *Salix* leaves, grass, *Thamnolia,* other lichens and moss. In wet conditions egg cooling coefficients increased for all materials. To account for the effect of different nest lining materials, we calculated an aggregated (weighted mean) nest lining material cooling coefficient based on the assumption that nest lining is dry for 2/3 and wet for 1/3 of the time.

An estimate of the combined effect of these three nest features (nest cup depth, lining depth, lining material) on nest insulation was derived by multiplying the proportional differences in egg cooling coefficient between the measured nest and an average pectoral sandpiper nest for each of the effects described above, with the value of C predicted from these same equations for a typical pectoral sandpiper nest. Egg cooling rates were predicted for a wind speed of 5 m/s, a value typical for our study area during the incubation period [25].

### Statistical Analyses

To analyse depth to frozen ground in relation to date we took into account that multiple observations per nest were carried out and used linear mixed effects models. Nest number was entered as a random term and day + day^2^ and species were entered as fixed effects. To test for differences in slopes between species, we also included interactions.

Nest measurements such as scrape depth, nest cup depth, nest lining depth were averaged per species and plotted against body mass for the different species. As we did not measure individual body mass for the owners of the individual nests, we used the mean body mass per species (measured during incubation, Schekkerman et al. 2004). Instead of using untransformed body mass, we applied an exponent of 0.73, to account for the allometric effect of size on species’ metabolic activity [30]. The relationship between nest measurements and body mass^0.73^ and between the effects of the different nest characteristics on the cooling coefficient was investigated using linear mixed effects models, with the different nest measurements as the fixed effects and species as the random effect. Depending on the graphical model validation an appropriate variance structure was chosen. All analyses were carried out in R [31].

## Results

### Breeding Site

Shorebird nests that were located on a slope were most often oriented towards the south, but sometimes also to the west, east or north side (Fig. 2). In curlew sandpiper and red phalarope a relatively large proportion of nests was found in flat lowland. Most dunlin nests were found on flat ridge tops. However, there was no difference between species in mean rank score of thermal favourability of nest sites (Kruskall-Wallis nonparametric ANOVA, H_5_ = 4.08, P = 0.54), and rank scores were not related to body mass^0.73^ (F_1,4_ = 0.16, P = 0.70). The distribution of nests of all species combined across the five habitat types, was however significantly different from that expected based on the available habitat, with a higher proportion of nests found in thermally favorable habitats. (χ^2^ = 51, df  = 4, P<0.001).

### Depth of Frozen Ground

The depth of the frozen ground was ca 5 cm at the start of breeding in late June and increased to >50 cm in late July (Fig. 3). The depth of frozen ground increased nonlinearly with the progressing season with a different intercept for the different species, but the rate of change did not differ between species (day: F_1,105_ = 1839.29, P<0.0001; day^2^: F_1,105_ = 33.855, P<0.0001; species: F_1,200_, P = 0.006; day.species: NS; day^2^.species: NS, Fig. 3). The intercept decreased in the order: Pacific golden plover, ruddy turnstone, dunlin, red phalarope, curlew sandpiper, little stint. However, the depth of frozen ground at egg laying did not correlate with body mass^0.73^.

### Nest Cup Depth and Scrape Depth

Nest cup depth varied between 1.5 and 7.0 cm, while scrape depth (depth of nest cup without the lining material) varied between 3.1 and 10.0 cm. The largest variation between nests was found in red phalarope. Nest cup depth was not correlated with body mass^0.73^ (F_1,4_ = 0.13, P = 0.737). Scrape depth tended to decrease with body mass^0.73^ but not significantly (F_1,4_ = 5.15, P = 0.085, Fig. 4 left). The measure for nest shape, nest cup depth/diameter1*diameter2, significantly increased with body mass^0.73^ (F_1,4_ = 32.30, P = 0.0047, Fig. 4 right): small species had narrow deep and large species had wide shallow nest cups.

### Lining Thickness and Material

**Figure 5:**
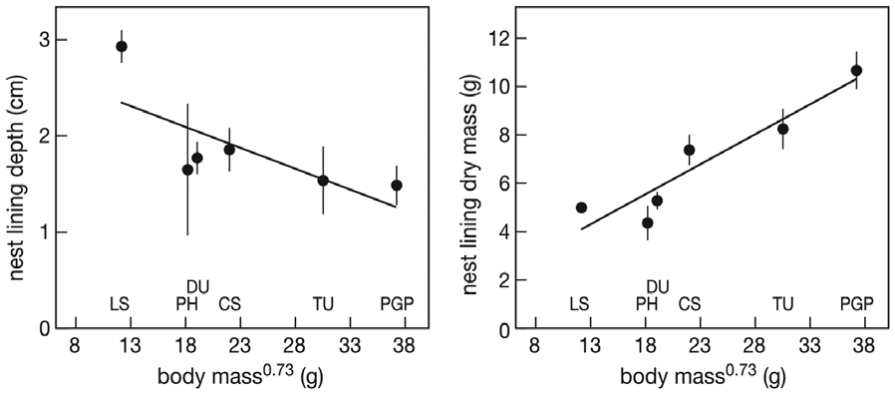
Nest lining depth (left) and nest lining dry mass (right) in relation to. body mass^0.73^. LS  =  little stint, PH  =  red phalarope, DU  =  dunlin, CS  =  curlew sandpiper, TU  =  turnstone, PGP  =  Pacific golden plover. Average and se values are represented for each species. The line represents the regression line.

**Figure 6:**
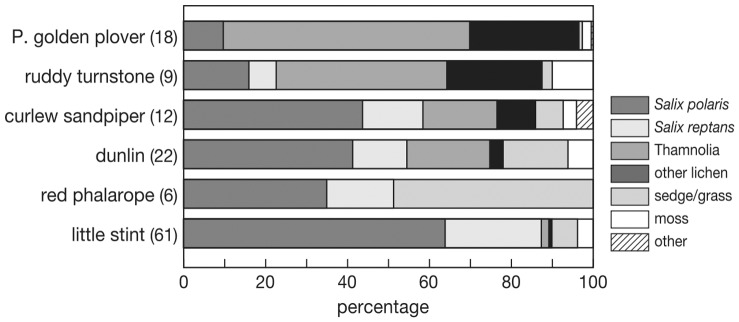
Nest lining material used by six different species with number of nests in brackets. LS  =  little stint, PH  =  red phalarope, DU  =  dunlin, CS  =  curlew sandpiper, TU  =  turnstone, PGP  =  Pacific golden plover.

The thickness of nest lining varied between 0.1 cm and 7.6 cm, was thickest in the smallest species and tended to decrease (but not significantly) with body mass^0.73^ (F_1,4_ = 5.89, P = 0.072, Fig. 5 left). Dry mas of the nest lining decreased with increasing body mass^0.73^ (F_1,4_ = 26.38, P = 0.0068, Fig. 5 right). Little stints nearly exclusively used willow leaves of the two species present, *S. reptans* and *S. polari*s (Fig. 6). This was also important nest material for dunlin, curlew sandpiper and red phalarope. Red phalarope was the only species that lined the nest with a large proportion of grass and sedges. Ruddy turnstone and Pacific golden plover preferred to line their nests with the lichen *Thamnolia vermicularis* supplemented with other lichens, willow leaves and a small fraction moss. Moss was used by all species in very small quantities, except by red phalarope.

### Composite Approximation of Egg Cooling Coefficient

**Figure 7:**
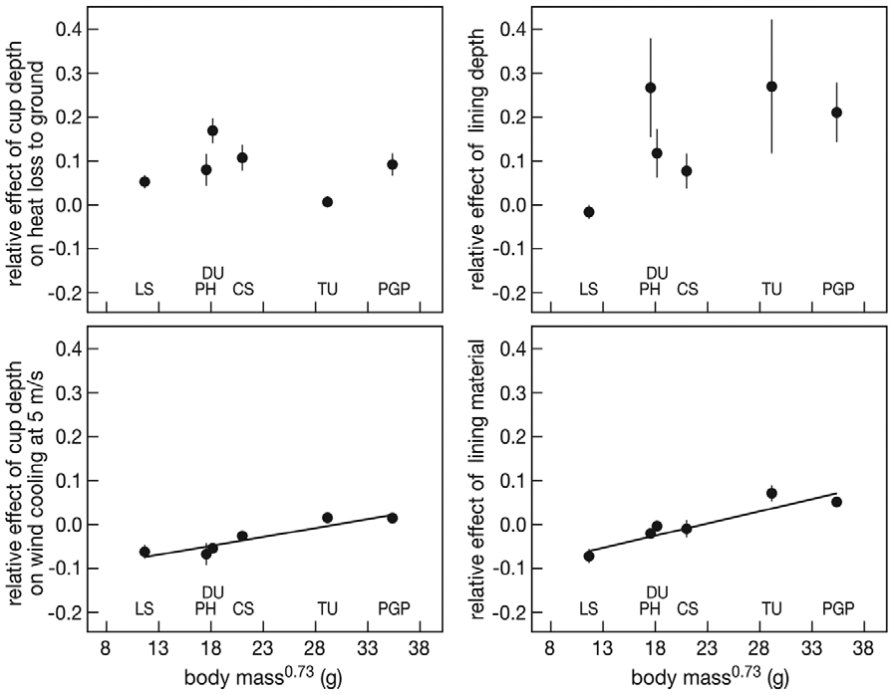
The relative contribution of cup depth on heat loss to the ground (upper left), of cup depth on wind cooling at 5 m/s (lower left), of lining depth (upper right)and of lining material (lower right) on egg cooling rates in relation to body mass. LS  =  little stint, PH  =  red phalarope, DU  =  dunlin, CS  =  curlew sandpiper, TU  =  turnstone, PGP = Pacific golden plover. Average and se values are represented for each species. The lines represent the regression lines.

The effect of nest cup depth on the proportion difference in cooling coefficient through heat loss to the ground was not correlated with body mass^0.73^ (F_1,4_ = 0.090, P = 0.778, Fig. 7 upper left). The relative sheltering effect of the nest cup at wind speed of 5 ms^−1^ on the cooling coefficient increased significantly with body mass^0.73^ (F_1,4_ = 34.23, P = 0.004, Fig. 7 lower left). The nest lining depth effect on egg cooling was uncorrelated to body mass^0.73^ (F_1,4_ = 5.087, P = 0.087, Fig. 7 upper right). The effect of nest material on the egg cooling coefficient increased significantly with body mass^0.73^ (F_1,4_ = 24.77, P = 0.008, Fig. 7 lower right).

These four separate effects were aggregated into one combined effect on egg cooling at a wind speed of 5 m/s, a value rather normal for this area in summer (Schekkerman et al. 2004, Fig. 8). The cooling coefficient thus predicted increased significantly with body mass^0.73^ (F_1,4_ = 16.079, P = 0.016), indicating that the contribution of the different adaptations to reduce heat loss is relatively larger in the smaller species.

**Figure 8:**
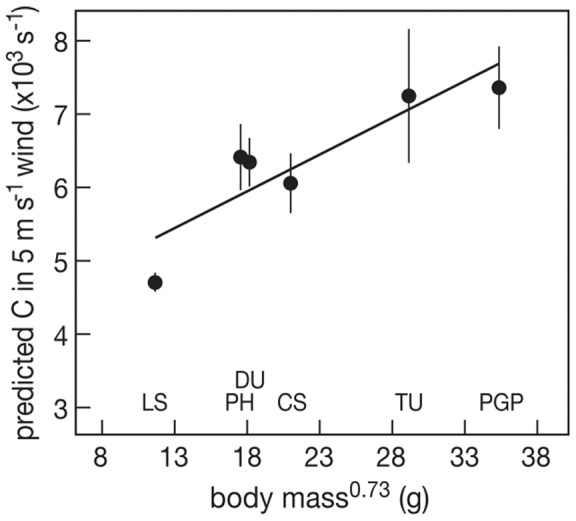
The predicted egg cooling coefficient in wind of 5 m/s in relation to body mass^0.73^. LS  =  little stint, PH  =  red phalarope, DU  =  dunlin, CS  =  curlew sandpiper, TU  =  turnstone, PGP  =  Pacific golden plover. Average and se values are represented for each species. The line represents the regression line.

## Discussion

### Nest Design

We measured characteristics of shorebird nests and found significant relations between nest shape, thickness and type of lining material and species body mass. These patterns result in a stronger reduction of heat loss from nests of small species compared to nests of larger species. The distance between the surface and frozen ground declined with date and was largest in the larger species. All species seemed to have a preference for south-facing slopes and selected the thermally favourable sites. This may be the result of the fact that south-facing slopes are cleared of snow earlier in the season and available for nest building. The smaller species had deeper and narrower nests than the larger species, a pattern which has been described in birds before [5]. Our estimates of the egg cooling coefficients predict that eggs in nests of the larger species cool down more rapidly and the different adaptations to reduce heat loss have a stronger effect in the smaller species. A difference in nest size and insulation related to body size was also observed in two species of arctic breeding geese [32].

### Egg Cooling Coefficient Calculations

Our estimates of egg cooling rates are based on extrapolation from the relationships derived in pectoral sandpiper nests using artificial eggs [18]. The thermal properties and measured heat loss rates of the artificial eggs that were used in [18] may deviate from the values in real pectoral sandpiper clutches. In our interspecies comparisons there was no correction for egg size, but egg cooling rates referred to the situation where eggs of the size of those of pectoral sandpipers would have been put in the nests of the different species. Given the comparisons of relative values used in this study, we are confident that any pattern shown up using extrapolated relationships, would also appear if real eggs had been used. However, small eggs cool down more rapidly than large eggs [5], therefore the relations found will probably decrease in strength if the size effect is taken into account.

### The Nest with and without the Incubating Bird

We calculated egg cooling rates for the situation when the bird is off the nest. Most of the time (81–87%) however, even uniparental incubators are on their nest [20]. In general, the smaller uniparental species leave the nest more often for shorter intervals than larger species, but total recess time does not differ between the species. Our estimates of egg cooling concerns the situation when the parent bird has left the nest and eggs cool down. But what happens when the bird is on the nest? If the parent returns to the nest the eggs need to be rewarmed. At the instant when the egg temperature reaches the steady state, the energy flow into the egg is the same as the energy flow going out of the egg. As [33] pointed out, at this moment the eggs are basically an extension of the bird’s body. Some of the benefits of nest construction as shown for the situation without the parent present, are likely also valid when a bird is incubating [34]. Both lining material and lining thickness still contribute to the insulative properties [35]. However the effect of wind cooling, acting through nest cup depth for the eggs in an open nest, will affect the incubating bird differently. But still the incubating bird will likely be better sheltered from the wind in deeper nest scrapes [8]. Birds may not only stay on the nest because it is beneficial for the development of eggs, but also to conserve energy, as time spent away from the nest generally costs more energy than incubating the eggs [11], [19].

### Lining Material

That nest insulation is apparently of importance to arctic shorebirds, particularly the smaller species, suggests that the supply of lining material may determine nest site choice and habitat suitability. The choice of nest lining material naturally depends on what material is available. Of the two *Salix* species of which dry leaves were used as lining, *Salix polaris* predominated, but was also the most common in the area. From the selection of materials found in shorebird nests, willow leaves had the best insulative properties. In the smaller species this was also the material that was used most. One material that retains warmth even better, down or feathers [18], [34], [36], [37], [38], [39], was never used in any of the shorebird nests. The reason for this is probably not the lack of availability (feathers can be taken from own plumage), but the fact that cooling coefficient of feathers is strongly increased in wet conditions. When wet, the insulative effect of feathers has been shown to be degraded from the best to the second worst in the row: feathers, *Salix* leaves, grass, lichen and moss [18], [40]. Considering that weather in the tundra is often humid and foggy, feathers are probably not as suitable here as in other areas (or in closed nests). Another reason to avoid using feathers is that they may attract predators through their smell [41].

The effect of lining depth was relatively important compared to other effects (Fig. 7). The thickness of nest lining showed considerable variation within individual nests of the same species (Fig. 5). Although we do not have the proper measurements to test this hypothesis, this individual variation might well be explained by differences in microclimates to which birds adapt the amount of lining. In an experiment where the amount of nest material was manipulated, the parents restored original amount of nest material both in nests where nest material was reduced and increased [42]. Parents apparently carefully balance the various costs and benefits of nest material use during incubation. Further evidence that birds adjust the amount of nest lining to environmental conditions is provided by [38], who describe that long-tailed tits *Aegithalos caudatus*, whose nests were provisioned with extra feathers, compensated for this by reducing the number of feathers they brought in themselves.

### Why don’t Large Waders Insulate their Nest Better?

Our analysis showed that the smallest species of shorebirds invested most in nest insulation. The smallest species in our sample also all happen to be uniparental species: little stint, red phalarope and curlew sandpiper, while the two largest species (Pacific golden plover, turnstone) are biparental. Dunlin is the only small species in our sample with a biparental mating system.

This makes it impossible to disentangle effects of the parental care system and body size on nest construction. The reason why the small uniparental species that face the highest energetic demands [11] try to optimise nest insulation seems obvious. Also from other studies it has been shown that nest insulation can have an important effect on incubation effort and hatching success [43]. So why do the larger biparental species not adopt this energy saving strategy and insulate their nests better?

First of all, the costs of a poor insulation may not be so high for larger species. Apart from an energetically more beneficial surface to volume ratio, they also produce larger eggs, that cool down slower than small eggs [44]. Furthermore the larger species in our sample are all biparental, which means the eggs are rarely left alone and incubation is near constant [45], [46]. This prevents the eggs from cooling down during foraging trips. Especially rewarming eggs upon return from a recess period elevates energy expenditure for the incubating parent [47], [48].

Secondly the benefit of a better nest insulation might not outweigh the costs associated with the extra effort. A deeper scrape needs more work excavating and the nest material has to be collected. Incidental observations in the field showed that most of the nest material is brought to the nest item by item. This can take considerable time and effort. Especially to collect large amounts of small willow leaves, the material with the best insulative properties, will require a substantial amount of time (e.g. little stint nests consisted of 1000–2000 leaves).

The larger species tended to nest in different habitat than the smaller species. Pacific golden plover and turnstone generally nested in drier tundra often characterised as frost-boiled tundra where lichens, bare soil, grass and herbs predominate [22]. Little stint, curlew sandpiper and dunlin nest in wetter habitat with more dry willows leaves present. Not all materials are equally abundant everywhere. Although it is impossible at this stage to distinguish cause from consequence, the smaller species could be restricted in their choice of nesting sites to habitat patches where the most profitable nest lining material can be obtained.

Arctic breeding shorebirds rely heavily on their extremely well-camouflaged eggs, and in most cases also plumage, that makes it very difficult for predators to find the nests. The use of local materials can improve the strong crypsis and this benefit may outweigh the benefits of a better insulating lining. The extreme of this trade-off between thermal properties and camouflage has resulted in a nest consisting of pebbles only, such as found in the Ringed Plover, a species co-occurring in the same area in low numbers. The lichen *Thamnolia* often used by Pacific golden plover and turnstone provides a much better camouflage in the habitat where these species breed than some of the better insulating materials.

Finally, biparental species tend to start breeding earlier than uniparental species [25], [49]. At the onset of spring the depth of frozen ground is still relatively close to the surface and making a deep scrape is simply impossible, or the cooling caused by the proximity of the ice outweighs the advantage of a deep scrape. By the time that uniparental species start nesting, the frost has retreated deep enough to be limiting the scrape depth.
